# Pharmacological effects of asiatic acid in glioblastoma cells under hypoxia

**DOI:** 10.1007/s11010-017-2965-5

**Published:** 2017-02-15

**Authors:** Flourina Kumar Thakor, Ka-Wai Wan, Philip John Welsby, Gail Welsby

**Affiliations:** School of Pharmacy and Biomedical Sciences, University of Central Lancashire, Preston, Lancashire PR1 2HE UK

**Keywords:** Glioblastoma, U87-MG, Asiatic acid, Hypoxia

## Abstract

Glioblastoma multiforme is the most common and malignant primary brain tumor in adults. Despite current treatment options including surgery followed by radiation and chemotherapy with temozolomide and cisplatin, the median survival rate remains low (<16 months). Combined with increasing drug resistance and the inability of some compounds to cross the blood–brain barrier, novel compounds are being sought for the treatment of this disease. Here, we aimed to examine the pharmacological effect of Asiatic acid (AA) in glioblastoma under hypoxia. To investigate the effects of AA on cell viability, proliferation, apoptosis, and wound healing, SVG p12 fetal glia and U87-MG grade IV glioblastoma cells were cultured under normoxic (21% O_2_) and hypoxic (1% O_2_) conditions. In normoxia, AA reduced cell viability in U87-MG cells in a time and concentration-dependent manner. A significant decrease in viability, compared to cisplatin, was observed following 2 h of AA treatment with no significant changes in cell proliferation or cell cycle progression observed. Under hypoxia, a significantly greater number of cells underwent apoptosis in comparison to cisplatin. While cisplatin showed a reduction in wound healing in normoxia, a significantly greater reduction was observed following AA treatment. An overall reduction in wound healing was observed under hypoxia. The results of this study show that AA has cytotoxic effects on glioma cell lines and has the potential to become an alternative treatment for glioblastoma.

**Electronic supplementary material:**

The online version of this article (doi:10.1007/s11010-017-2965-5) contains supplementary material, which is available to authorized users.

## Introduction

Glioblastoma multiforme (GBM), a grade IV astrocytoma, is the most aggressive and malignant form of glioma. It accounts for 12–15% of all intracranial tumors and is characterized by molecular heterogeneity and resistance to therapy [1, 2]. Patients with high-grade glioma have a poor prognosis and median survival is <5 years depending on tumor grade, cytogenetic analysis, age, and performance status at the time of diagnosis [2]. Radiotherapy and/or concomitant chemotherapy with adjuvant temozolomide (TMZ) have become the standard of care in GBM [3, 4]. Temozolomide is a prodrug that undergoes metabolic activation following oral administration; thus its in vitro use is restricted [5]. Cisplatin is used to treat recurrent glioma; however, its clinical use is limited due to cellular resistance and dose-dependent toxicity in normal tissue [6–8].

The growth/development and internal organization of all animals depends on the maintenance of oxygen homeostasis and depending on the organ size, function, and tissue location, a wide range of oxygen tension is encountered (0.002–10%; normal brain 16–21%) [9, 10]. A drop in oxygen partial pressure activates transcription of hypoxia inducible factor (HIF) leading to changes in cellular metabolism due to transcription of a wide array of genes such as *EGFR* (epidermal growth factor receptor), *VEGFR* (vascular endothelial growth factor receptor), and *GLUT-1* (glucose transporter-1) [9, 11, 12]. Hypoxia further promotes the malignant phenotype of cancer cells, and hypoxic cancer cells often exhibit enhanced resistance to chemotherapy and radiation. Hypoxia is a predominant factor in GBM and plays an important role in tumor growth and progression [13]. Thus, it is important to establish the cytotoxicity of anti-cancer agents under hypoxia.

Asiatic acid (AA) is a pentacyclic triterpenoid extracted from *Centella asiatica*, native to countries including India and Sri Lanka [14, 15]. It displays a low side-effect profile and exhibits cytotoxicity against glioblastoma, breast, liver, and colon cancers [16]. In addition, evidence shows that AA can cross the BBB and it has been reported to induce apoptotic cell death in human hepatoma and malignant glioma cells [17, 18].

In this study, the pharmacological effects of AA under normoxia (21% O_2_) and hypoxia (1% O_2_) have been investigated. To calculate drug efficacy, results have been compared to the chemotherapeutic agent, cisplatin.

## Materials and method

### Cell culture

U87-MG (human Grade IV glioblastoma) and SVGp12 (human Fetal glial) cell lines obtained from American Type Culture Collection (ATCC, UK) were maintained in a 37 °C humidified atmosphere containing 5% CO_2_ in Eagles Minimum Essential Media (EMEM), supplemented with 10% fetal bovine serum, 2 mM L-glutamine, 1% non-essential amino acids, and 1mM sodium pyruvate (Lonza, UK). For hypoxic experiments (1% O_2_, 5% CO_2_, 94% N_2_), cells were seeded under normoxia (5% CO_2_, 95% air) and allowed to adhere for a minimum of 2 h before transferring to hypoxia using an InvivO_2_ 400 hypoxia workstation (Baker Ruskinn, UK). Cells were allowed to acclimatize to hypoxia overnight prior to treatment.

### Cell viability assay

Cisplatin and AA (#P4394 and #A2612; Sigma Aldrich, UK) concentration–response curves in U87-MG and SVGp12 cell lines were performed under normoxia. Cells were seeded at a density of 10^3^ cells/well in 96-well plates and cultured for 24 h prior to drug treatment (10^− 7^–10^− 4^ M). Cell viability was measured at 24-, 48-, and 72-h post-treatment using PrestoBlue^®^ (Thermo Fisher Scientific, UK) by measuring fluorescence at an excitation/emission of 535/612 nm after 1 h incubation at 37 °C using a GENios Pro plate reader (Tecan, UK). All treatments were performed in triplicate and a concentration–response analysis was performed (GraphPad Prism 5, GraphPad Software Inc., USA) to determine EC_50_ and EC_25_ concentrations for subsequent assays.

### Cell proliferation assay

Cell proliferation was measured using carboxyfluorescein diacetate succinimidyl ester (CFDA-SE) dye (Cambridge Bioscience, UK). Cells were seeded at a density of 10^5^ cells/well in 6-well plates and 24 h later labeled with CFDA-SE (5 µM in 1X PBS) for 30 min. Fresh medium was added and cells were treated with EC_25_ concentrations of cisplatin (2 μM) and AA (30 µM) for 24, 48, and 72 h under normoxia or hypoxia. Cells were harvested by trypsinization and flow cytometry was performed on a Guava® easyCyte 12HT benchtop flow cytometer (Merck Millipore, UK). A total of 10,000 gated events representing the single cell population were analyzed to determine peak CFDA-SE fluorescence (488 nm) for each treatment population.

### Cell cycle analysis

U87-MG cells were seeded at a density of 10^5^ cells/well in 6-well plates and treated with 2 µM cisplatin and 30 µM AA, the EC_25_ determined by the cell viability assay, for 24, 48, 72, and 120 h under normoxia and hypoxia. Cells were harvested by trypsinization, fixed in 70% ethanol, and stained using 1X PBS containing RNase (250 µg/ml) and propidium iodide (5 µg/ml) for 30 min. Analysis was performed on a Guava^®^ easyCyte 12HT benchtop flow cytometer (Merck Millipore, UK). A total of 10,000 gated events representing the single cell population were analyzed to determine DNA content by fluorescence intensity (488 nm) for each treatment population.

### Western blot analysis

U87-MG cells were treated for 48 h with the EC_50_ concentration of cisplatin (10 µM) and AA (50 µM) as determined by the cell viability assay. Cells were harvested by scraping into 250 µl of radio-immunoprecipitation assay buffer (RIPA) with protease and phosphatase inhibitor cocktails (Sigma Aldrich, UK) and solubilization achieved by 1 hr incubation on a rotating wheel at 4 °C. Protein content was normalized by BCA assay, samples (40 μg) separated by SDS–PAGE (12% bis-acrylamide), and transferred to PVDF nylon membranes (GE Healthcare Life Sciences, UK) using a wet transfer kit (Bio-Rad, UK). Membranes were blocked for 1 h at room temperature with 5% skimmed milk in Tris-buffered saline-0.1% Tween 20 (TBS-T), then incubated with cyclin B1 antibody (1:1000 dilution; Cell Signaling Technology, UK) in TBS-T overnight at 4 °C with gentle shaking. Primary antibody detection was performed following incubation with HRP-conjugated secondary antibody (1:2000 dilution; Cell Signaling Technology, UK) at room temperature for 1 h and visualized by enhanced chemiluminescence (Amersham prime ECL Prime-chemiluminescent agent; Bio-Rad, UK) on a Bio-Rad Molecular Imager ChemiDoc™ XRS + System with Image Lab™ Software, UK. Following densitometry, bands were expressed as fold change compared to non-treated normoxic control.

### Apoptosis assay

Cells were seeded at a density of 10^5^ cells/well in 6-well plates and treated with 10 μM cisplatin and 50 μM AA. EC_50_ was determined by the cell viability assay, under normoxia and hypoxia. Following 24, 48, and 72 h of treatment, cells were harvested by trypsinization and labeled using the Dead Cell Apoptosis Kit with Annexin-V/Alexa Fluor^®^ 488 and Propidium Iodide (Invitrogen/Life Technologies™, UK) as per the manufacturer’s instructions. Analysis was performed by flow cytometry on a FACSAria (BD Bioscience, UK) and Guava^®^ easyCyte 12HT benchtop flow cytometer (Merck Millipore, UK). A total of 10,000 gated events were analyzed and the relative fluorescence (488 nm) of propidium iodide plotted against Annexin-V/Alexa Fluor^®^ to identify healthy and apoptotic cell populations.

### Wound healing/migration assay

An in vitro scratch assay was performed using U87-MG cells under normoxia and hypoxia. Cells were seeded at 4.5 × 10^4^ cells/well in a 24-well plate, incubated overnight, and the resulting cell monolayer was scraped in a single line using a sterile toothpick to create a scratch and the media was replaced to remove dislodged cells. Cells were treated with 2 μM cisplatin and 30 μM AA, the EC_25_ concentration determined by the cell viability assay. The scratch was imaged immediately, and after 18 h of incubation, using a modified Zeiss Cell Observer imaging system using a Zeiss Plan-Apo 20 × 0.8 NA air objective (Zeiss, UK). Scratch diameter was measured using an average of six points on a minimum of three separate images per treatment using the Zeiss Zen desk software. Results were expressed as percentage closure vs non-treated control.

### Statistical analysis

Statistical analysis was performed using one-way and two-way ANOVA with Bonferroni’s post hoc test as described in GraphPad Prism software version 5 (GraphPad Software, GraphPad Software Inc., USA). Significant differences were accepted for *p* < 0.05 and have been represented as *p < 0.05, **p < 0.01, and ***p < 0.001. Results were expressed as mean ± SEM of three individual experiments.

## Results

### Dose and time responses for the viability effect of asiatic acid in vitro

**Fig. 1:**
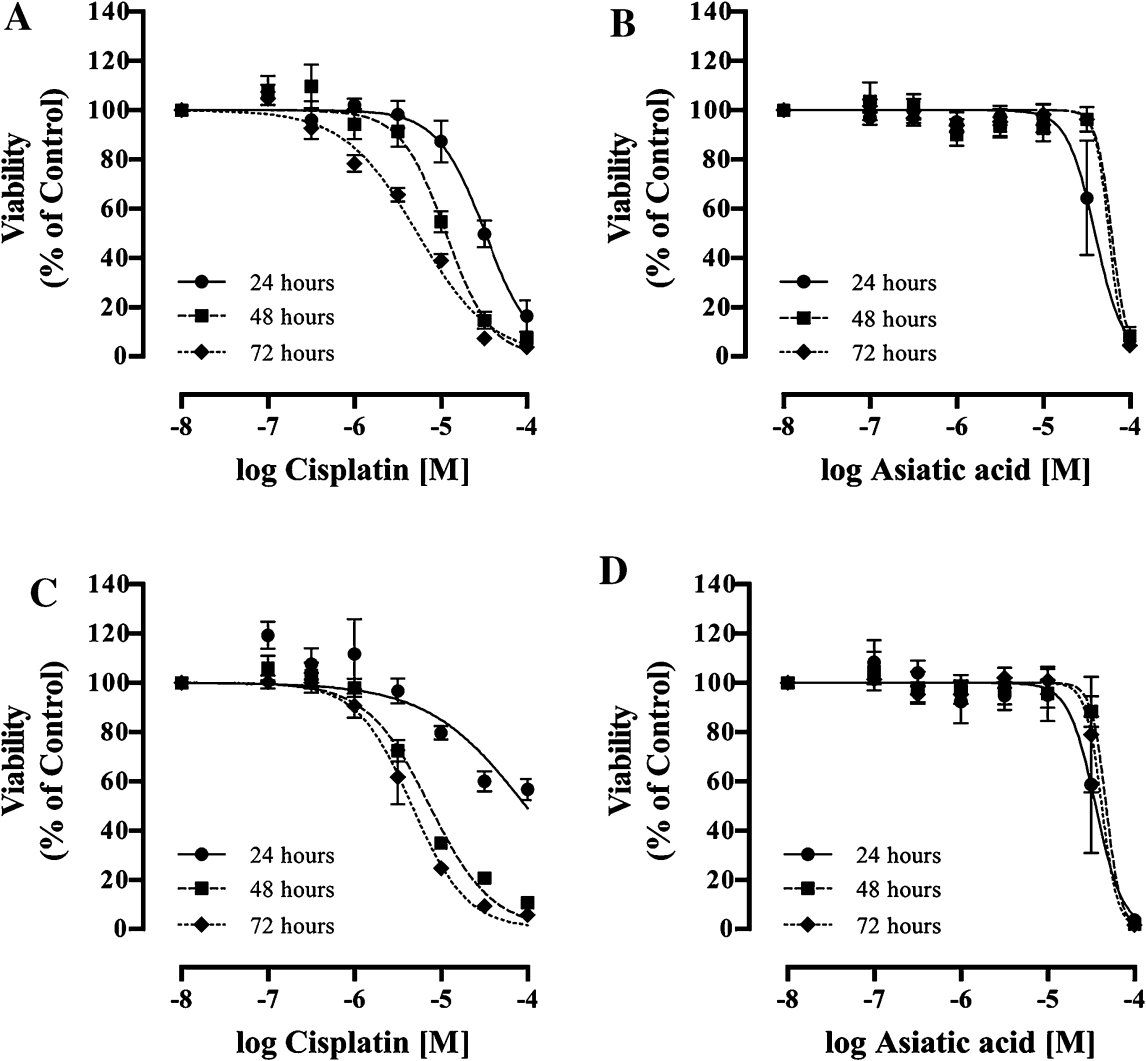
Concentration- and time-dependent changes in cell viability following cisplatin and AA treatment at 24, 48, and 72 h of (**a** and **b**) the SVGp12 and (**c** and **d**) the U87-MG cell lines. Data represent mean ± SEM of three independent experiments. Statistical significance determined by ANOVA with Bonferroni’s post hoc test (p < 0.05)

The potential of cisplatin and AA to affect U87-MG human glioblastoma cell viability was investigated in vitro and compared to the non-cancerous SVGp12 cell line. A concentration- and time-dependent decrease in cell viability was observed for both cisplatin and AA (Fig. 1). While no overall significant difference was found between each of the treatments (*p* > 0.05), significant differences were observed between EC_50_ values (Table 1). Following 24 h of treatment in the U87-MG cell line, the EC_50_ value for AA was significantly lower than that of cisplatin (44 ± 20 vs. 97 ± 11 μM respectively; *p* < 0.01). However, following 48 and 72 h of treatment, the EC_50_ value for cisplatin was significantly lower than that of AA in U87-MG cells (7.4 ± 0.4 vs. 47.4 ± 3.0 μM; *p* < 0.05 and 4.7 ± 1.0 vs. 59 ± 17.0 μM; *p* < 0.01 respectively).

**Table 1:** Cell viability EC_50_ values for cisplatin and AA under normoxia following 24, 48, and 72 h of treatment

Normoxia	Cisplatin (μΜ)			Asiatic acid (μΜ)		
	24 h	48 h	72 h	24 h	48 h	72 h
SVG p12	33.8 ± 7.9	11.2 ± 1.4	5.4 ± 0.5	50.3 ± 18.0	66.6 ± 7.6	56.6 ± 3.6
U87-MG	97.0 ± 11.7	7.4 ± 0.4	4.7 ± 1.0	44.3 ± 20.2	47.4 ± 3.1	59.0 ± 17.3

Data represent mean ± SEM of three independent experiments. Statistical significance determined by ANOVA with Bonferroni’s post hoc test (*p* < 0.05)

As cell viability can be influenced by multiple factors, it was decided to examine the effects of AA on cell proliferation and apoptosis following 48 h of treatment using either the EC_50_ or EC_25_ as determined by the cell viability assays. Additionally, as the efficacy of treatment can be affected by oxygen levels in the tumor microenvironment, subsequent assays were performed under both normoxia (21% O_2_) and hypoxia (1% O_2_).

### Effect of asiatic acid on U87-MG cell proliferation

**Fig. 2:**
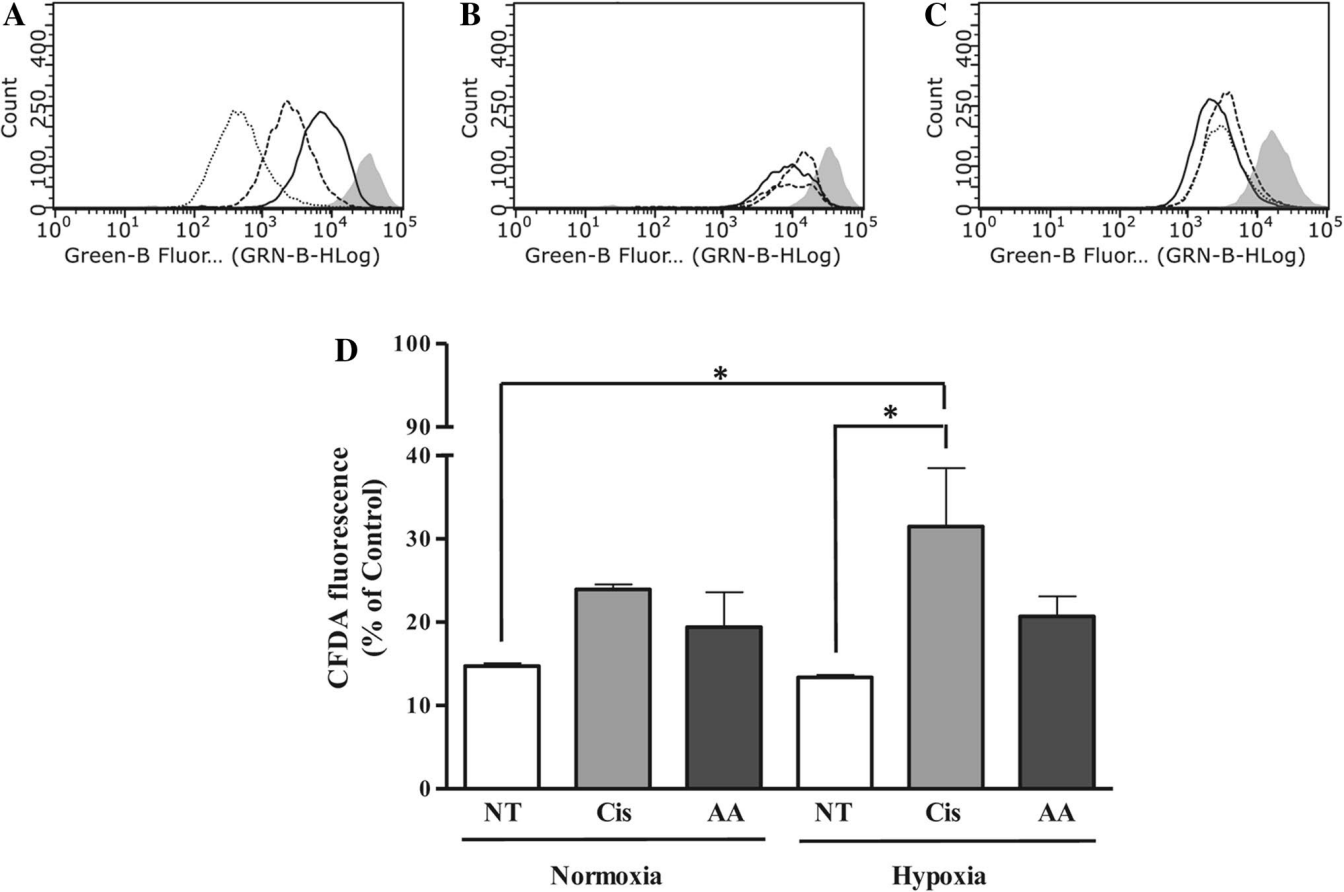
Effect of cisplatin and AA treatment on cell proliferation in U87-MG cells. Representative flow cytometric plots showing CFDA-SE fluorescence intensity in non-treated controls (**a**) immediately after staining with CFDA (*gray* fill) and at 24 (*straight lines*), 48 (*dashed lines*), and 72 (*dotted lines*) hours and under normoxia; following 48 h of incubation for control (*straight lines*), cisplatin (*dashed lines*) and AA (*dotted lines*) treatments with non-treated control immediately after incubation with CFDA (*gray fill*) under normoxia (**b**) and hypoxia (**c**). Graphical representation of cell proliferation as determined by mean CFDA-SE fluorescence intensity (**d**) following 48 h of treatment under normoxia and hypoxia. *NT non-treated, Cis Cisplatin, AA Asiatic acid*. Data represent mean ± SEM of three independent experiments. Statistical significance determined by One-Way ANOVA with Dunnett’s post hoc test (*p* < 0.05)

To investigate the effect of AA on U87-MG cell growth, a proliferation assay was performed. Following staining with CFDA-SE, under normoxia, flow cytometric analysis showed a decrease in CFDA fluorescence in untreated cells over 72 h (Fig. 2a). Cells were treated with cisplatin and AA under normoxia and hypoxia using the EC_25_ concentration determined by the cell viability assay. A significant decrease in proliferation was noted in cisplatin-treated U87-MG cells under hypoxia at 48 h when compared to the non-treated normoxic (31.5 ± 7.0 vs. 14.7 ± 0.4%) and hypoxic (31.5 ± 7.0 vs. 13.4 ± 0.3%) controls (Fig. 2b, d, p < 0.05). AA treatment showed no significant decrease in proliferation under normoxia or hypoxia vs either control or cisplatin treatment (Fig. 2c, d). This result indicated that the effect of AA on cell viability was not due to a significant decrease in cell proliferation. Similarly, AA did not reduce SVGp12 cell proliferation (see Online Resource 2).

### Cell cycle arrest in U87 cells following cisplatin and asiatic acid treatment

**Fig. 3:**
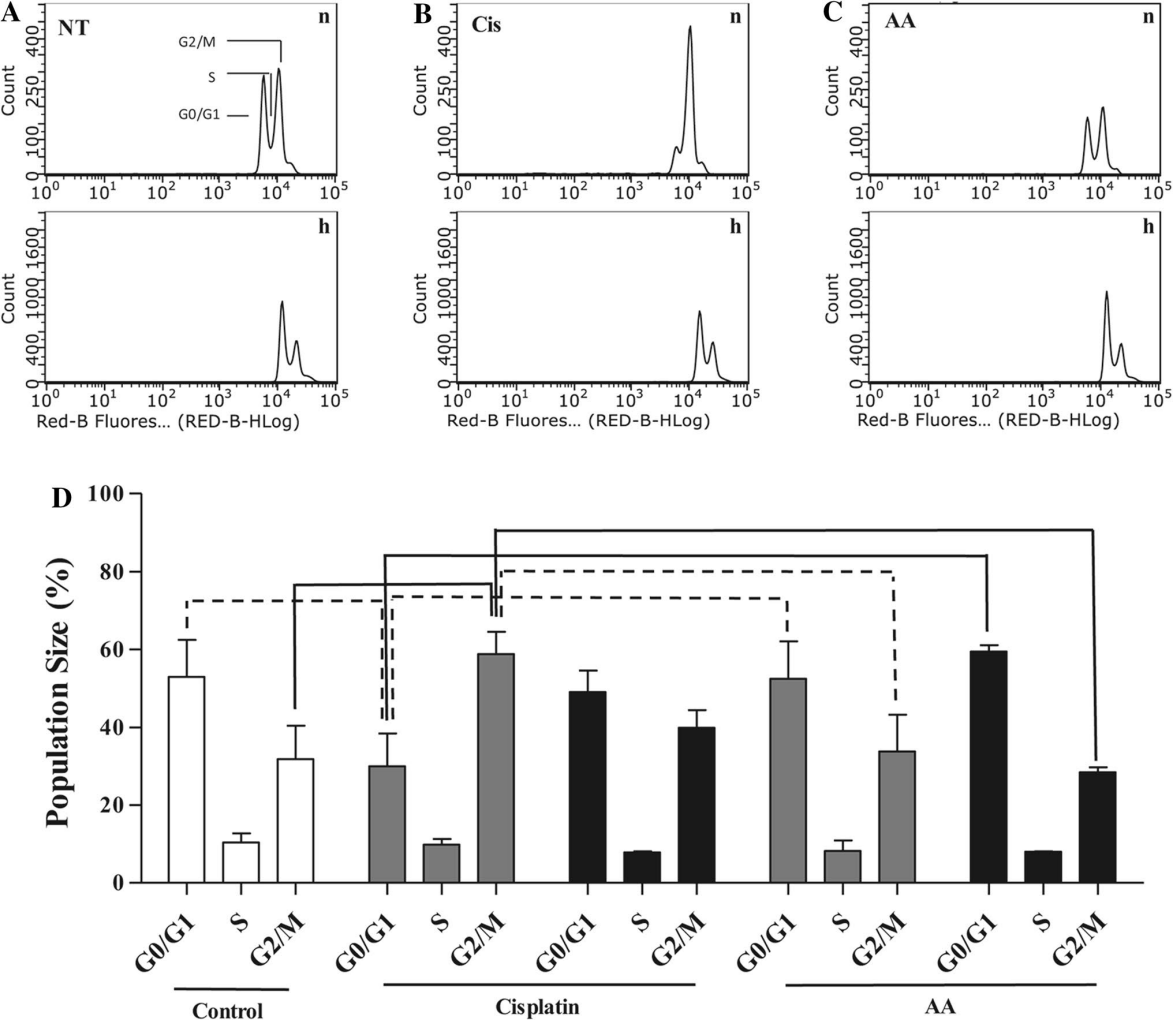
Effect of cisplatin and AA treatment on cell cycle in U87-MG cells. Representative flow cytometric plots showing PI fluorescence intensity following 48 h incubation for (**a**) non-treated, (**b**) cisplatin, and (**c**) AA treatments under normoxia (n) and hypoxia (h). (**d**) Cell cycle analysis of U87-MG cells following 48 h of cisplatin treatment and AA treatment (*white: control, gray: normoxia, black: hypoxia*). Data represent mean ± SEM of three independent experiments. Statistical significance determined by ANOVA with Bonferroni’s post hoc test (*p* < 0.05, *dotted line; p* < 0.01, *solid line* comparisons)

To further establish the effects of cisplatin and AA on cell growth, cell cycle arrest was measured by flow cytometry. Normoxic U87-MG cells treated with the EC_25_ of cisplatin showed a significant decrease in the proportion of G0/G1 phase cells compared to non-treated controls (Fig. 3a, b, and d; 30.0 ± 8.4% vs. 53.0 ± 9.6%; *p* < 0.05) with a concomitant increase in the proportion of cells in G2/M phase compared to non-treated controls (Fig. 3a, b, and d; 58.9 ± 5.7% vs. 31.8 ± 8.7%; *p* < 0.01). AA had no significant effect on U87-MG cell cycle distribution under either normoxia or hypoxia compared to control (Fig. 3c, d). Neither AA, nor cisplatin treatment at EC_25_ induced apoptosis (see Online Resource 1).

**Fig. 4:**
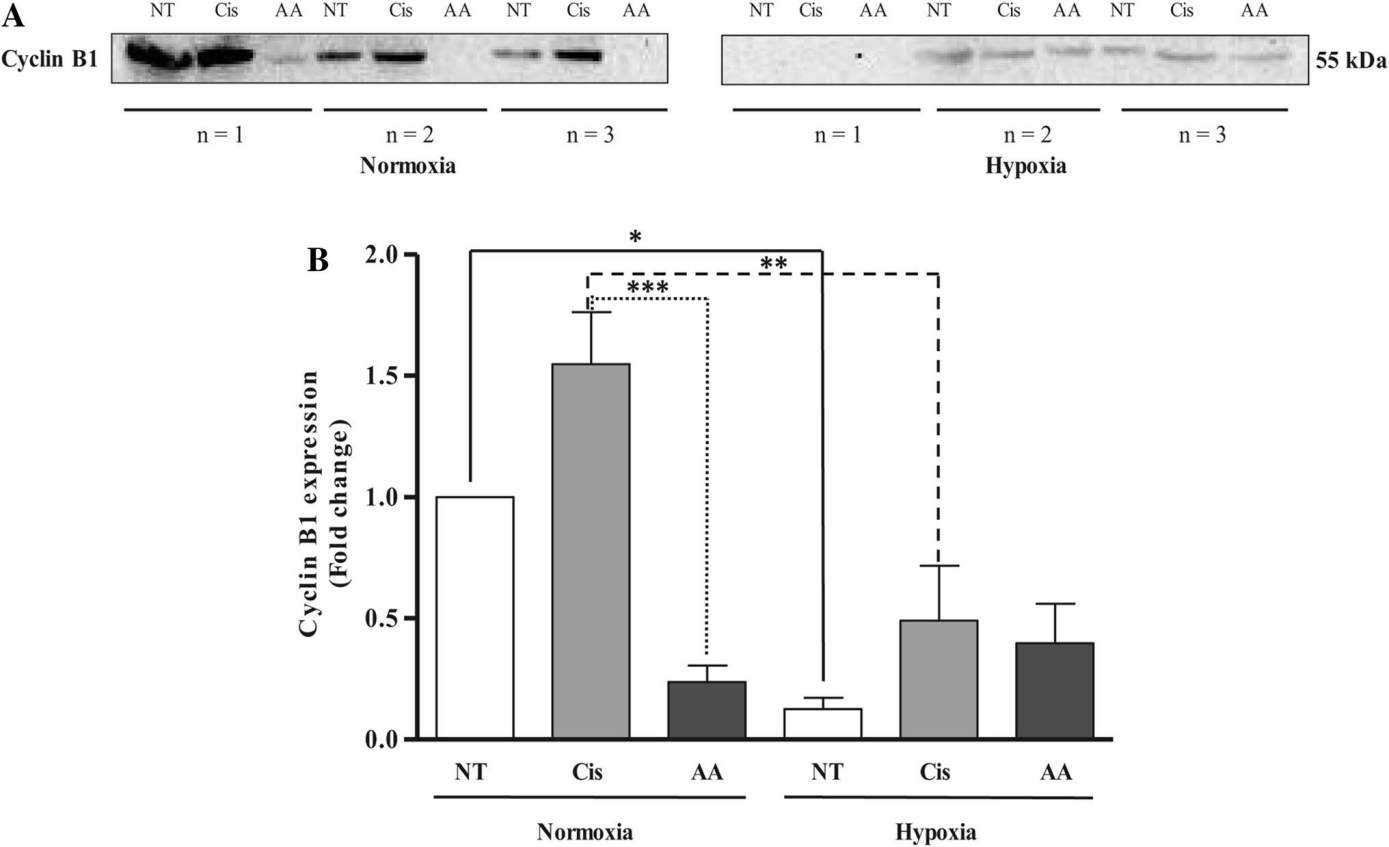
Western blot analysis for cyclin B1 expression in cisplatin- and AA-treated U87-MG cells under normoxia and hypoxia (**a**); graphical representation of fold change in cyclin B1 expression compared to non-treated normoxia control (**b**). *NT non-treated, Cis cisplatin, AA Asiatic acid*. Data represent mean ± SEM of three independent experiments. Statistical significance determined by ANOVA with Bonferroni’s post hoc test (*p* < 0.05)

Western blot analysis of cyclin B1 expression showed an increase in cyclin B1 levels in U87-MG cells following 48-hour cisplatin treatment under normoxia (Fig. 4b), correlating with the cell cycle arrest in G2/M in these cells. While the cyclin B1 levels following AA treatment were not significantly different from control under normoxia, they were significantly lower than following cisplatin treatment (Fig. 4b 0.2^4^ ± 0.07-fold vs. 1.55 ± 0.22-fold respectively; *p* < 0.001). Under hypoxia, both non-treated (0.13 ± 0.05-fold; *p* < 0.05) and cisplatin-treated (0.49 ± 0.23-fold; *p* < 0.01) cyclin B1 levels were significantly lower than their normoxic equivalents (Fig. 4b).

### Induction of apoptosis in U87-MG cells

**Fig. 5:**
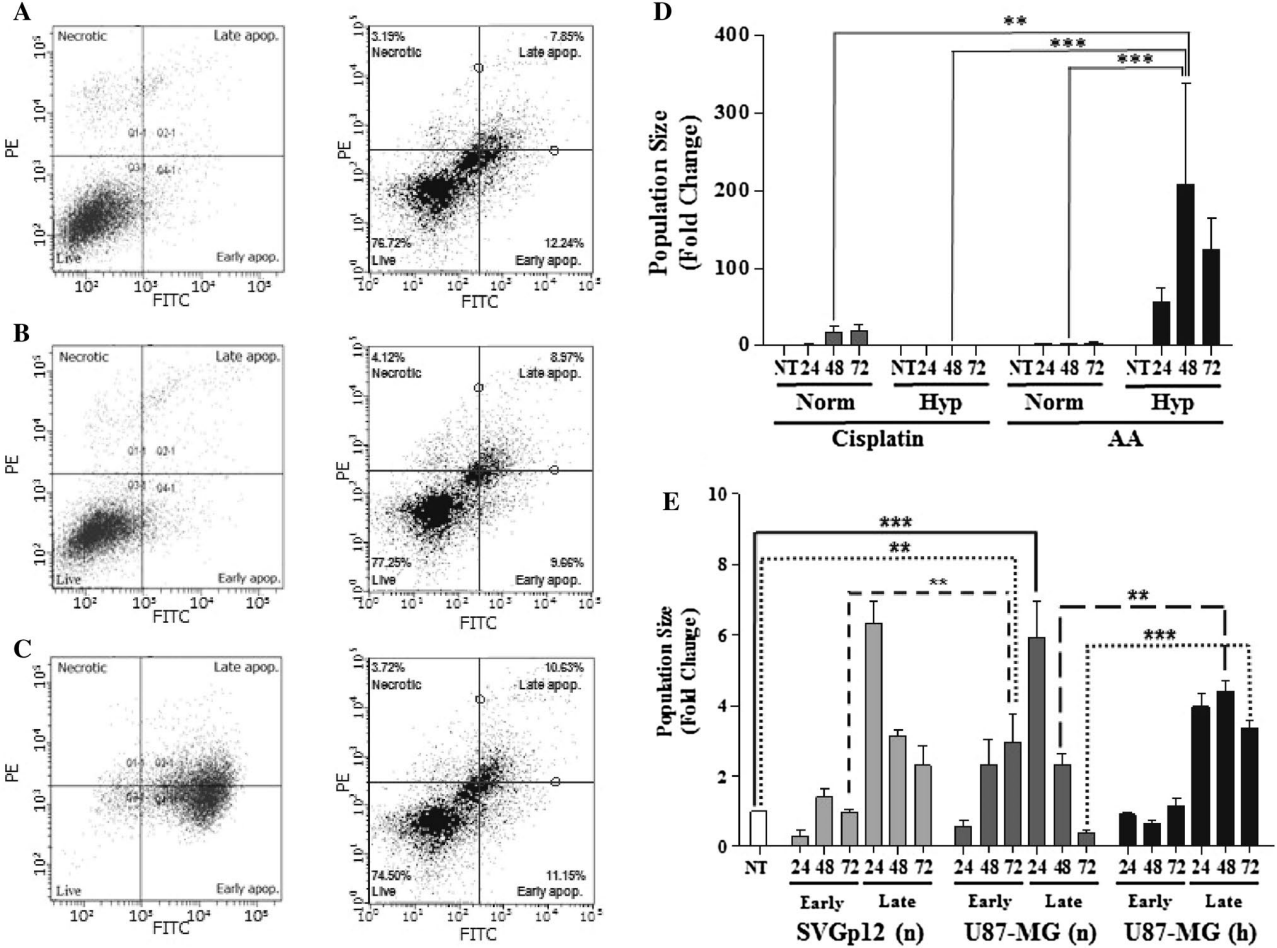
Induction of apoptosis following 48-h treatment of the U87-MG cell line under normoxia (n) and hypoxia (h) with cisplatin or AA. Representative dot plots showing fluorescence intensity of PI vs Annexin-V staining in (**a**) non-treated, (**b**) cisplatin, (**c**) AA. Relative proportion of total apoptotic U87-MG cells following 24, 48, and 72 h of cisplatin and AA treatment under normoxia and hypoxia (**d**), data normalized to normoxic non-treated cells. Relative proportions of early and late apoptotic cells following 24, 48, and 72 h of AA treatment (**e**) under normoxia and hypoxia, data normalized to non-treated SVGp12 cells. Data represent mean ± SEM of three independent experiments. Statistical significance determined by ANOVA with Bonferroni’s post hoc test (*p* < 0.05)

Having shown that cell proliferation and cell cycle are significantly regulated by cisplatin but not by AA treatment, induction of apoptosis was examined over 72 h following treatment of cells with cisplatin and AA using the EC_50_ concentration determined by cell viability assays. The total apoptotic population was determined by combining the cell population staining positively for Annexin-V alone (early apoptosis) with that staining positively for both Annexin-V and PI (late apoptosis). The total apoptotic population following 48 h of drug treatment with AA under hypoxia was significantly greater than the equivalent treatment under normoxia (Fig. 5d; 207 ± 131-fold vs. 3.1 ± 0.3-fold respectively; *p* < 0.001), in addition to both the normoxic (Fig. 5d 16.8 ± 9.6-fold; *p* < 0.01) and hypoxic (Fig. 5d 0.8 ± 0.1-fold; *p* < 0.001) populations following 48 h of cisplatin treatment.

Early-phase apoptosis significantly increased following 72 h of AA treatment under normoxia in U87-MG cells when compared to non-treated controls (Fig. 5 e 3.0 ± 0.8-fold; *p* < 0.01), a level of apoptosis that was significantly greater than the equivalent population in SVGp12 cells (Fig. 5 e 1.0 ± 0.1-fold; *p* < 0.01). AA treatment also induced a significant increase in late-phase apoptosis compared to non-treated control following 24 h of treatment under normoxia (Fig. 5e 5.9 ± 1.0-fold; *p* < 0.001). Interestingly, the proportion of late-phase apoptosis induced by AA treatment at both 48 h (Fig. 5 e 4.4 ± 0.3-fold; *p* < 0.01) and 72 h (Fig. 5 e 3.3 ± 0.2-fold; *p* < 0.001) under hypoxia was significantly greater than the equivalent treatment under normoxia (Fig. 5 e 48 h, 2.3 ± 0.3-fold; 72 h, 0.4 ± 0.1-fold).

### Wound healing/migration assay in U87-MG cells

**Fig. 6:**
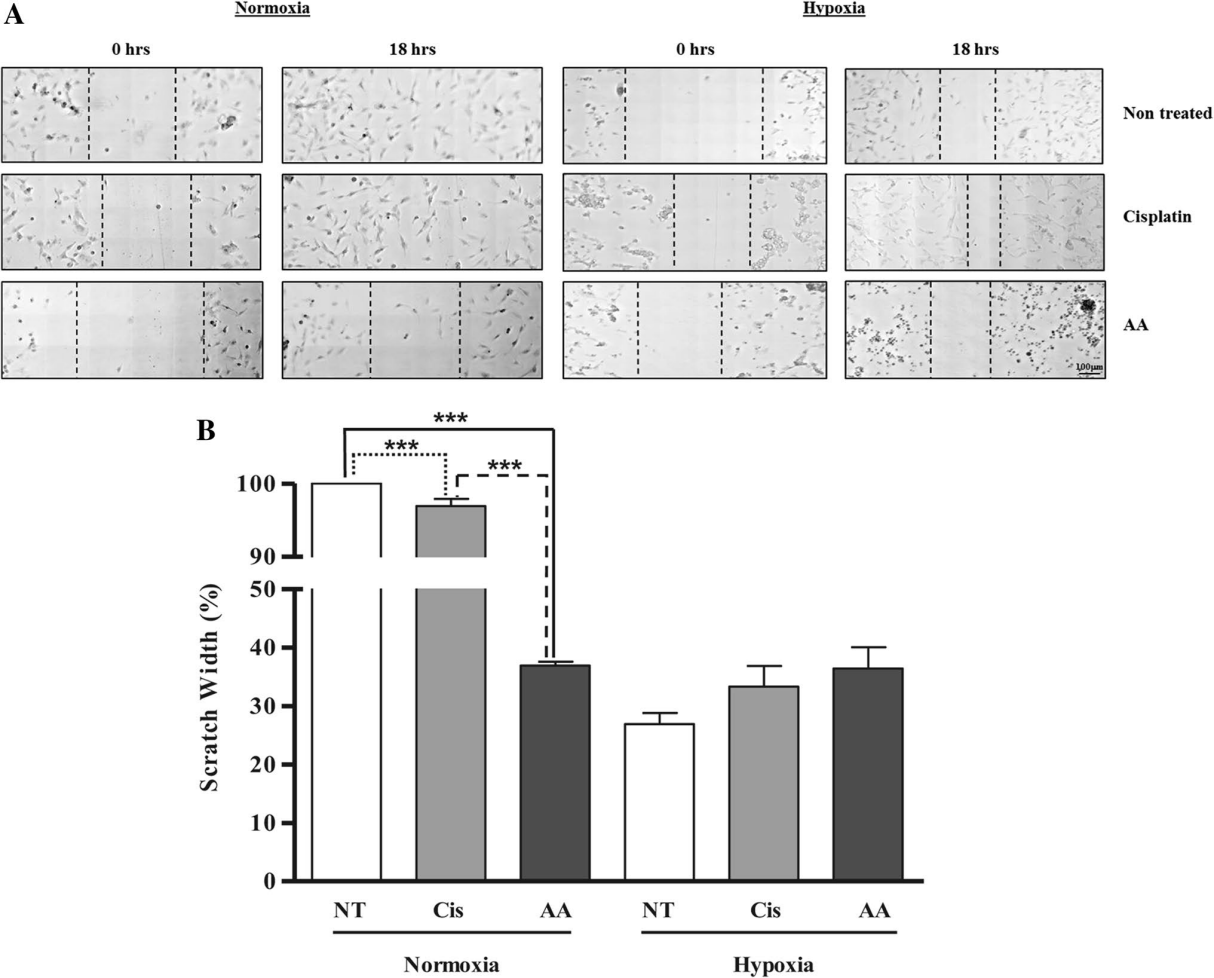
Inhibition of wound healing/migration by cisplatin and AA treatment in U87-MG cells under normoxia and hypoxia. (**a**) Representative microscopy images showing initial scratch and subsequent width following 18 h of incubation. (**b**) Graphical representation of wound healing following 18 h of treatment under normoxia and hypoxia with results expressed as a percentage of initial wound width. *NT non-treated, Cis cisplatin, AA Asiatic acid*. Data represent mean ± SEM of three independent experiments. Statistical significance determined by One-Way ANOVA with Bonferroni’s post hoc test (*p* < 0.05)

Finally, cell migration was examined using the wound healing assay to determine any effect of cisplatin or AA under normoxia and hypoxia. Under normoxia in non-treated cells, the scratch completely closed during the 18-h incubation (Fig. 6a, b). Treatment with cisplatin produced a small but significant reduction in wound healing compared to control (Fig. 6b, 97.0 ± 1.0%; *p* < 0.001), with AA producing a larger reduction in cell migration under the same condition compared to control (Fig. 6b, 36.9 ± 0.7%; *p* < 0.001). Importantly, the reduction in migration produced by AA was significantly greater than the reduction produced by cisplatin treatment under normoxia (*p* < 0.001). Hypoxia itself significantly reduced wound healing in control cells when compared to normoxia (Fig. 6b, 26.9 ± 1.9% vs. 100% respectively; *p* < 0.001). Under hypoxia, neither drug treatment with cisplatin (33.3 ± 3.5%), nor AA (36.4 ± 3.7%) significantly changed cell migration when compared to hypoxic control.

## Discussion

Cisplatin (cis-diamminedichloroplatinum) is a potent chemotherapeutic drug widely used for cancer treatment [7]. The clinical use of cisplatin includes treatment of sarcomas; ovarian and cervical cancers; cancers of the bone, soft tissue, and blood; and pediatric solid tumors [19]. Cisplatin enters cells by passive diffusion and produces its cytotoxicity *via* DNA intercalation inducing apoptosis and changes in cell cycle [6, 20–23]. However, cisplatin efficacy is reduced under hypoxia [24], and its unfavorable toxicological profile characterized by nephrotoxicity, neurotoxicity, nausea, vomiting, and immunosuppression limit its clinical usefulness [6, 25]. Additionally, the absorption of cisplatin into the perifocal tumor is hindered by the presence of the BBB [26]. In contrast, AA has been established as a potential therapeutic agent in many cancer types, has a low risk of severe side effects, anti-angiogenesis properties [27], and has shown to cross the BBB [28]. Thus, the main aim of this study was to investigate the anti-cancer effects of AA on glioblastoma cells in vitro under normoxia and hypoxia.

Both cisplatin and AA produced a decrease in U87-MG cell viability in a time- and concentration-dependent manner. As cisplatin exerts its cytotoxicity by forming DNA lesions, this mechanism of action delayed reductions in cell viability until after 48 h of treatment [6, 29]. AA demonstrated greater cytotoxicity in the U87-MG cell line than in SVGp12, a finding that correlates with a recent study which also observed consistently lower cell viability in U87-MG cells compared to SVGp12 cells [30]. It has been suggested that reduced cell viability following AA treatment is due to endoplasmic reticulum stress as a result of activated GRP-78 and an increase in intracellular calcium level, which decreases the mitochondrial membrane potential, leading to cell death [17, 18].

Due to DNA intercalation, cisplatin is known to disrupt the cell cycle [31, 32], an effect replicated in this study where a reduction in the rate of proliferation of U87-MG cells and cell cycle arrest in the G2/M phase was observed under normoxia. Cyclin B1 and cyclin-dependent kinase 1 (CDK1) specifically regulate cell’s entry into mitosis, and an increase in cyclin B1 expression observed by western blotting confirmed cell cycle arrest in the G2/M phase of cisplatin-treated cells under normoxia [33]. Although AA has formerly been reported to induce a G2/M phase arrest in RPMI 8226 cells [16] and S-G2/M arrest in MCF-7 and MDA-MB-231 breast cancer cell lines [34], in U87-MG cells under normoxia or hypoxia, AA did not show any significant changes in cell proliferation or cell cycle distribution. This is in agreement with previous studies that used glioblastoma cell lines and found that AA inhibits cell viability mainly via cell death, but their effects on cell proliferation or cell cycle have not been reported [30].

Decrease in oxygen supply to cells leads to biochemical changes that either result in cell death or adaptation to hypoxia regulated by HIF-1α [35]. This effect is reportedly due to the cyclin-dependent kinase inhibitor p27^Kip1^ that inhibits the activation of cyclin E-Cdk2 or cyclin D-Cdk4 complexes thus controlling cell cycle progression at G1 phase [36]. Hypoxia results in a slower cell cycle progression, whereas moderate hypoxia (e.g., 1%) induces pre-DNA-synthetic (pre-S-phase) arrest in cells, while cells in the other phases of cell cycle, progress to late G1 phase before they arrest [37]. This transient arrest in G1 phase has been described as a possible mechanism to protect cells from proceeding into the S phase, where they are more sensitive to hypoxia-related DNA injuries [38]. Cisplatin treatment of U87-MG cells under hypoxia decreased cell proliferation; however, the number of cells arrested in G2/M phase of cell cycle following hypoxic cisplatin treatment was lower than under normoxia. AA treatment under hypoxia did not produce significant changes in cell proliferation or cell cycle; however, a greater population of cells was observed in the G0/G1 phase of cell cycle. The lower levels of cyclin B1 expression under hypoxia in comparison to normoxia along with an increase in cell populations at G0/G1 phase confirms that cells were not arrested in G2/M phase of cell cycle.

Under normoxia, a time-dependent increase in apoptosis was observed in U87-MG cells following cisplatin treatment; a finding in agreement with previous studies conducted using cisplatin [30, 39]. Cisplatin exerts an additive cytotoxic effect on cells as they proliferate and a prolonged exposure to cisplatin leads to apoptotic cell death [31]. Cell proliferation is an important factor for the cytotoxic effects of cisplatin due to its mechanism of action. As hypoxia results in slower cell proliferation, the total apoptotic population of cells following hypoxic cisplatin treatment was lower than normoxic. This finding has been confirmed in other studies where a reduced efficacy of cisplatin under hypoxia was observed [24, 40].

Early and late apoptotic cells are distinguished by a loss of membrane integrity and propidium iodide staining in late apoptotic cells [41]. A large proportion of SVGp12 and U87-MG cells were observed in late apoptosis, confirmed by Annexin-V and PI binding, following AA treatment under both normoxia and hypoxia. Interestingly, a time-dependent shift in the proportion of cells in late apoptosis back into the early apoptotic quadrant was observed for normoxic SVGp12 and U87-MG cells following AA treatment, possibly suggesting a loss of efficacy of AA treatment over time following a single-drug treatment. This is not a surprising result as AA has been shown in vivo to generate a large number of phase I metabolites, and additionally a number of cytochrome p450 enzymes such as CYP2C9 are known to be highly expressed in brain tumors [42, 43]. This effect was not observed following hypoxic AA treatment and a large number of late apoptotic cells were observed which did not change significantly over 72 h. AA causes DNA fragmentation and also induces a loss of phosphatidylserine symmetry that results in phosphatidylserine being exposed on the outer cell membrane [17, 34]. AA-mediated cell death in U87-MG cells involves caspase-9 and caspase-3 activation and intracellular calcium release that triggers a biochemical cascade resulting in calpain activation and subsequent cell death due to breakdown of the cellular architecture [17, 34, 44]. As AA cytotoxicity is not dependent on cell proliferation, an overall increase in apoptotic population of AA-treated cells was observed under hypoxia in comparison to cisplatin.

The in vitro scratch assay is a method of studying cell migration. GBMs are characterized by an aggressively invasive phenotype that leads to infiltration into the surrounding brain tissue, a characteristic which makes it difficult to eliminate with gross resection and chemotherapy, thus any treatment resulting in a decrease of the invasive phenotype may have therapeutic potential. When a cell monolayer is wounded or scratched, it responds to the cell–cell disruption by increasing growth factors at the wound site and ultimately results in healing of the wound through migration and cell proliferation [45]. Microscopic analysis following normoxic and hypoxic AA treatment showed rounded cells, a characteristic of apoptotic cell death [46]. A larger proportion of apoptotic cells were observed under hypoxia than under normoxia, suggesting again that AA does not regulate cell proliferation or cell cycle but instead exerts its cytotoxicity via inducing cell death. As cells proliferate slowly under hypoxia and cells may undergo cell cycle arrest or senescence, an overall reduction in wound healing was also observed vs normoxic control [47]. As cisplatin mainly exerts its cytotoxicity after 48 h of incubation, the lack of efficacy of cisplatin on wound healing under normoxia may be explained in part by the shorter time scale used for these experiments.

Results of this study collectively demonstrate the cytotoxic potential of AA on in vitro glioblastoma cells. Although AA did not regulate cell proliferation and cell cycle progression of SVGp12 and U87-MG cells, a higher proportion of cells underwent apoptosis under hypoxia. In contrast to cisplatin treatments under hypoxia, a greater efficacy of AA on induction of cellular apoptosis and a significant delay in wound healing were observed. With its reduced side effect profile and increased time course for cytotoxicity under both normoxia and hypoxia, these data suggest that AA has the potential to become an alternative therapy for the treatment of glioma.

## Electronic supplementary material

Below is the link to the electronic supplementary material.

Supplementary material 1 (EPS 1592 KB)

Supplementary material 2 (EPS 1239 KB)
